# Impact of the 4th of August Beirut explosion mass casualty incident on a university hospital microbial Flora

**DOI:** 10.1186/s12879-023-08818-4

**Published:** 2024-01-04

**Authors:** Zeinab Roumieh, Hanine Mansour, Rawad Abi Assaad, Hani Dimassi, Rola Husni, Sanaa Zoghby, Jacques E. Mokhbat

**Affiliations:** 1 School of Pharmacy department of pharmacy practice, Lebanese American University, Byblos, Lebanon; 2 School of Medicine, Lebanese American University, Beirut, Lebanon; 3 School of Pharmacy, Lebanese American University, Beirut, Lebanon; 4 School of Pharmacy department of pharmaceutical sciences, Lebanese American University, Byblos, Lebanon; 5 Infection control, Lebanese American University Medical Center-Rizk Hospital (LAUMC-RH), Beirut, Lebanon

**Keywords:** Multi-drug resistant microorganisms (MDRO), Hospital acquired infections (HAI), Infection control, Blast-related injuries, Combat zones, Antimicrobial resistance (AMR), Surgical infection prevention

## Abstract

**Background:**

Following the Beirut explosion, our university hospital received at least 350 casualties. Subsequently, infection control standard practices were compromised. Concerns for Multi-Drug Resistant Organisms (MDROs) infections in injured patients and a resulting hospital outbreak were raised. The objectives of the study were to compare the rate of hospital growing MDROs 6 months before and 6 months after the Beirut explosion, to identify emerging microorganisms and to evaluate the change in surgical infection prevention practices.

**Methods:**

This is a retrospective chart review of patients with hospital acquired infections (HAI) admitted to the hospital before and after the Beirut explosion. The study was conducted between February 4, 2020 and January 4, 2021. Excluded patients were those transferred from other hospitals and those with community acquired infections. The primary outcome was to identify the rate of growing MDROs post explosion. The secondary outcomes were identifying antibiotics used for surgical prophylaxis in patients requiring surgeries and patients diagnosed with a HAI. Therefore, patients were divided in three groups. Control group included patients admitted with explosion-related injuries on that same day. Patients admitted and between February 4 and August 4 and diagnosed with HAI were compared to those admitted post August 4 with explosion-related HAI and to patients diagnosed with non-explosion-related HAI between August 4 and January 4, 2021. An estimated rate of 18-22% MDRO was needed to achieve a statistical significance with 80% power and 0.05 α. Pearson Chi square test was used to analyze the primary outcome.

**Results:**

A total of 82 patients with 150 cultures were included in this study. Data showed an increase in the rate of MDRO after the explosion with 37.1% of the cultures taken before the explosion and 53.1% after the explosion (*p* = 0.05). When comparing the types of HAI in both groups, culture sites were significantly different between pre- and post-explosion patients (*p* = 0.013). However, both groups had similar types of microbes (*p* = 0.996) with an increase in candida related infections.

**Conclusion:**

These findings confirmed that the Beirut explosion impact on antimicrobial resistance was similar to combat zone incidence, where an increase in MDROs rate such as *Escherichia coli* (E.Coli) and *Stenotrophomonas maltophilia*, in addition to the increase in candida related infections.

## Background

On August 4th 2020, a massive explosion caused by a large amount of ammonium nitrate stored at the port of Beirut, the capital of Lebanon, has led to hundreds of deaths, thousands of injuries, billion US$ in property damage, and leaving thousands of people homeless [1]. This unexpected devastation flooded all hospitals in Beirut, which was quickly filled beyond capacity [2].

Minutes after the explosion, the Lebanese American University Medical center –Rizk Hospital (LAUMC-RH), a 138-bed university hospital, was among centers near the site of the explosion received around 350 casualties with different types of injuries, therefore overwhelming the hospital’s capacity and emergency resources. The infection control practices at the hospital, including hand hygiene and aseptic practices in the operating rooms were compromised. Such practice could have possibly led to contamination of hospital surfaces. In the literature, this was reflected by the positive threshold of nosocomial infections (NIs) per departments; 90% in surgery, 87.5% in emergency departments and 75% in operating rooms [3]. In addition, conflict injures have been associated with multidrug resistant organisms (MDRO) hospital outbreak including *Acinetobacter baumannii calcoaceticus* complex (ABC), multidrug resistant *Pseudomonas aeruginosa*, and *Klebsiella pneumoniae* [4, 5]. According to the CDC (Centers for Diseases Control and Prevention), MDROs are defined as microorganisms, predominantly bacteria, such as methicillin resistant *staphylococcus aureus* (MRSA), vancomycin resistant *enterococci* (VRE) that are resistant to one or more classes of antimicrobial agents [1, 6]. The overuse or miuse of antibiotics, which is common in the emergency settings during natural disasters or wars has led to the appearance of new strains of MDROS and is among the top 10 threats to global health [7–9]. For instance, multidrug-resistant gram-negative bacteria such as *Pseudomonas aeruginosa* and carbapenem-resistant *K. pneumoniae*, mainly due to multiple bacterial genetic mutations, form a major threat to the public health system with a high rate of mortality [10, 11].

Also, infections associated with explosions and combat-related injuries have been associated with two types of contamination: early and late contamination [12]. Any wound due to war or explosion is considered contaminated at the time of injury. In general, it is assumed that late contamination is associated with MDROs due to the increased use of broad-spectrum antimicrobial, surgeries, emergency department visit, and ventilation associated infections [12]. Adding to that, the high turnover of healthcare and staff personnel and the admission of patients without proper screening on admission, increases the risk of contamination and MDROs emergence [12].

Therefore, to minimize combat or explosion-related infections and the emergence of MDROs, the Infectious Diseases Society of America and the Surgical Infection Society established a guideline discussing preventative measures that should be taken as soon as patients are transferred to the hospital. These measures include the following: “rapid drainage and coverage of the wound with sterile dressing, especially if those associated with bone damage; fractures should be stabilized before transfer to surgical rooms; patients planned for surgery should be transferred to the operation room as soon as possible; point-of injury antimicrobials should be administered to patients with unpredictable time of surgery transfer as soon as possible.” [13] Hence, we hypothesize that this breach in the basic standards precautions for infection control on the day of the explosion will result in a change of our institution flora with a possible isolation of new MDROs [14, 15].

The aim of this study is to evaluate the change in the hospital flora by comparing the rate of hospital growing MDROs 6 months before the Beirut explosion to 6 months after, and to identify emerging microorganisms and to evaluate the change of antimicrobial surgical prophylactic practices.

## Methods

This is a retrospective chart review of patients with hospital acquired infections (HAI) admitted to the hospital before and after the 4th of August Beirut explosion in 2020. The study was conducted over a period of 11 months; from February 4, 2020, until January 4, 2021. All patients diagnosed with HAI identified from infection control records were included. Patients with colonization were also included to assess the impact of the explosion on the acquired flora from the hospital setting. We excluded all patients transferred from other hospitals and those with community acquired infections. The primary outcome was the rate of growing MDROs post explosion in a university hospital. The secondary outcome was the change of antibiotics used for surgical infection prevention post-explosion. To identify the changes in the microbial flora among those admitted before and after the explosion, patients were divided in three groups. The first group (group 1) included all patients admitted to the hospital in the 6 months period prior to the explosion and developed HAI. The second group (group 2) were all patients admitted on 4th of August or later with explosion-related injuries and diagnosed with MDRs or developed HAIs during their hospital stay, those should serve as the control group. However, group 2 was overlooked since no cultures were taken on the day of the explosion or could not be assessed because the majority of the cultures were negative due to antibiotics use post-explosion. The third group (group 3) included patients admitted to the hospital post 4th of August due to explosion related injuries but not admitted earlier and developed HAI. Also, this group of patients received antibiotics post explosion without obtaining cultures. This has led to negative cultures due to antibiotics use post-explosion. Group 3 contains all patients admitted to the hospital after the explosion up until 6 months later (February 4th, 2021) and developed HAI. Hence, our main comparison in the study of MDRO will take place between groups 1 and 3. This poses a limitation in the study with the lack of an explosion-related control group.

An estimated rate of 18-22% MDRO is needed to achieve a statistical significance with 80% power and α 0.05. Pearson Chi square was used to analyze the primary outcome. McNemar chi square using binominal adjustment was used to analyze the number of candida species infection and its fluconazole resistance before and after the explosion.

The Lebanese American University Institutional Review Board with exempt status approved the study. The data was inserted on an excel sheet with the appropriate coding, then analyzed using IBM SPSS Statistics for Windows, Version 21.0.

## Results

The study retrieved 40 patients pre-explosion and 42 patients post-explosion with HAIs. Researchers studied more than 150 HAI and colonization antibiograms. The data showed an increase in the rate of MDRO post explosion with 37.1% MDRO pre-explosion versus 53.1% post explosion (*p* = 0.05). Even though a significant difference was noted, both groups showed a significant difference in the rate of previous MDRO colonization (45.0% pre-explosion vs 46.5% post-explosion, *p* = 0.890). It is important to note that despite having a higher MDRO rate post-explosion, the history of antibiotic use in patients pre-explosion was higher than post-explosion (82.1% vs 58.1%, *p* = 0.019). The rate of broad-spectrum empirical antibiotic use was similar in both groups with rates of 91.2% of antibiotics being broad-spectrum pre-explosion and 88.8% post-explosion (*p* = 0.626).

When comparing the rates of surgical interventions in both groups, no statistical significance was noted (30% pre-explosion vs 14% post-explosion, *p* = 0.076).

When comparing the types of HAI before and after the explosion respectively, we noticed that the type of infections prevalent in each group were different. (Table 1) The difference is approaching significance (*p* = 0.054). Only wound infection showed a statistical difference between pre and post explosion.

**Table 1:** The prevalence of different types of HAI before and after the explosion

Type of Infection	Pre-August 4th Beirut Explosion Group N (%)	Post-August 4th Beirut Explosion Group N (%)	*P*-value
SSI	6 (10.5)	4 (5.3)	
HAP	7 (12.3)	7 (9.2)	
CA-UTI	17 (29.8)	12 (15.8)	
Wound	5 (8.8)	1 (1.3)	^*^
VAP	17 (12.3)	13 (17.1)	
Central lines	2 (3.5)	10 (13.2)	
HA-UTI	2 (3.5)	7 (9.2)	
Colonization	11 (19.3)	20 (26.3)	
CSF	0 (0.0)	1 (1.3)	
Blood Stream	0 (0.0)	1 (1.3)	
Total	57 (100)	76 (100)	0.054^^^

*SSI* surgical site infections, *HAP* hospital acquired pneumonia, *CA-UTI* catheter-associated urinary tract infections, *HA-UTI* hospital-acquired urinary tract infections, *CSF* cerebrospinal fluid infection*s*

^^^ Exact *p*-value using Fisher-Freeman-Halton exact test

^*^ Indicates statistical difference between the 2 proportions using post-hoc analysis

Culture sites were significantly different between pre- and post-explosion patients (*p* = 0.013) (Table 2). For example, cerebrospinal fluid (CSF) cultures were only observed in the post-explosion group (0.0% vs 1.2%).

**Table 2:** The difference in culture sites between both groups

Culture Site	Pre-August 4th Beirut Explosion Group N (%)	Post-August 4th Beirut Explosion Group N (%)	*P*-value
Urine	27 (42.2)	26 (32.1)	
Blood	5 (7.8)	17 (21.0)	
Sputum	19 (29.7)	32 (39.5)	
Wound	13 (20.3)	5 (6.2)	
CSF	0 (0.0)	1 (1.2)	
Total	64 (100)	81 (100)	0.013

Both groups had similar types of microorganisms (p = 0.996). The rates of gram-positive bacteria, gram-negative bacteria and fungi were comparable: 17.1% vs 17.3% for gram-positive organisms, 60.0% vs 60.5% for gram-negative organisms and 22.9% vs 22.2% for fungi.

**Fig. 1:**
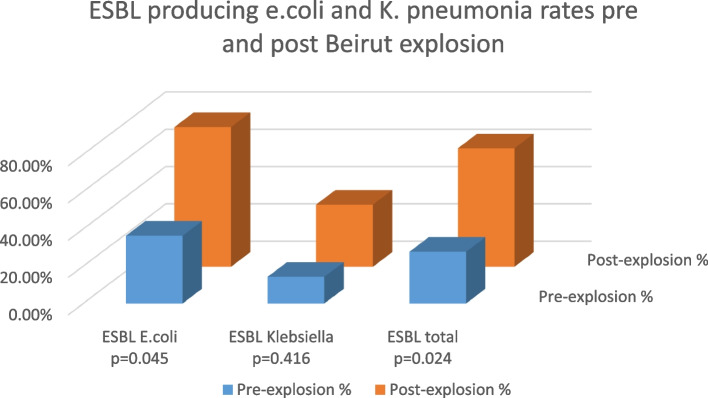
ESBL producing *E.coli* and *K. pneumonia* rate pre and post Beirut explosion

The doubling in the incidence of *Stenotrophomonas maltophilia* post-explosion (8.8%) compared to pre-explosion (4.3%) is noted. In addition, there is a remarkable increase in MDRO *Escherichia coli* (*E.coli*) emergence post-explosion (*p* = 0.045). The difference in MDRO *Klebsiella* was not statistically significant (*p* = 0.416). However, the total ESBL was higher post-explosion compared to pre-explosion with 63.6% vs 27.8% (*p* = 0.024) (Fig. 1).

Concerning fungal infections, *Candida albicans* became more prevalent post-explosion than pre-explosion (11.3% versus 5.7% respectively) (Table 3). It is important to note the appearance at our medical center of new candida species post-explosion such as *Candida parapsilosis*, *Candida krusei*, and *Candida kefyr*.

**Table 3:** Rates of different microorganisms in each group

Microorganism	Pre-August 4th Beirut Explosion Group N (%)	Post-August 4th Beirut Explosion Group N (%)	*P*-value
*Staphylococcus aureus*	1 (1.4)	2 (2.5)	
*Coagulase negative staphylococci*	5 (7.1)	6 (7.5)	
*Enterococcus faecalis*	3 (4.3)	2 (2.5)	
*Enterococcus faecium*	2 (2.9)	1 (1.3)	
*Escherichia coli*	11 (15.7)	16 (20.0)	
*Klebsiella pneumonia*	7 (10.0)	6 (7.5)	
*Klebsiella oxytoca*	1 (1.4)	0 (0.0)	
*Enterobacter cloacae*	6 (8.6)	4 (5.0)	
*Other Enterobacter*	0 (0.0)	1 (1.3)	
*Citrobacter freundii*	0 (0.0)	2 (2.5)	
*Citrobacter koseri*	2 (2.9)	0 (0.0)	
*Proteus mirabilis*	3 (4.3)	1 (1.3)	
*Morganella morganii*	0 (0.0)	1 (1.3)	
*Serratia spp*	1 (1.4)	1 (1.3)	
*Pseudomonas aeruginosa*	8 (11.4)	7 (8.8)	
*Stenotrophomonas maltophilia*	3 (4.3)	7 (8.8)	
Acinetobacter baumanii/ anitra	0 (0.0)	1 (1.3)	
*Candida albicans*	4 (5.7)	9 (11.3)	
*Candida glabrata*	7 (10.0)	2 (2.5)	
*Other Candida*	4 (5.7)	8 (10)	
*Alcaligenes xylosoxidans*	1 (1.4)	0 (0.0)	
*Burkholderia cepacia*	0 (0.0)	1 (1.3)	
*Enterococcus gallinarum*	0 (0.0)	1 (1.3)	
*Acinetobacter haemolyticus*	0 (0.0)	1 (1.3)	
*Total*	70 (100)	80 (100)	0.456

When comparing antibiograms, a notable increase in resistance to many antibiotics was noted without a significant difference compared to the pre-explosion period. However, some antimicrobials such as amoxicillin-clavulanate and imipenem showed a significant increase in resistance post-explosion (*p* = 0.008 and 0.012) respectively. Differences are represented in Table 4.

**Table 4:** Antimicrobials resistance rate difference between both groups

Antimicrobial	Pre-August 4th Beirut Explosion Group *N* (%)	Post-August 4th Beirut Explosion Group *N* (%)	*P*-value
Penicillin	13 (72.2)	9 (90.0)	0.272
Oxacillin	8 (80.0)	8 (100.0)	0.180
Ampicillin	30 (75.0)	40 (90.9)	0.051
Amoxicillin-clavulanate	15 (42.9)	22 (55.0)	0.008
Piperacillin	12 (37.5)	19 (57.6)	0.105
Piperacillin- tazobactam	4 (12.9)	8 (22.9)	0.204
Cephalothin	13 (43.3)	22 (95.7)	< 0.001
Cefuroxime	12 (40.0)	8 (50.0)	0.515
Cefoxitin	14 (50.0)	23 (76.7)	0.042
Ceftriaxone	11 (31.4)	20 (50.0)	0.103
Ceftazidime	10 (23.3)	22 (43.1)	0.043
Cefepime	6 (18.2)	13 (37.1)	0.082
Ceftaroline	2 (28.6)	–	–
Ceftolozane-tazobactam	6 (17.1)	1 (9.1)	0.517
Aztreonam	7 (25.0)	4 (36.4)	0.478
Imipenem	8 (22.9)	7 (63.6)	0.012
Meropenem	9 (25.7)	6 (54.5)	0.075
Ertapenem	5 (13.9)	11 (28.2)	0.131
Gentamicin	17 (31.5)	19 (31.1)	0.640
Amikacin	5 (15.2)	1 (9.1)	0.612
Chloramphenicol	2 (33.3)	4 (50.0)	0.533
Colistin	7 (21.2)	7 (20.0)	0.902
Tetracycline	23 (52.3)	11 (40.7)	0.345
Tigecycline	4 (9.5)	0 (0.0)	0.057
Erythromycin	7 (63.6)	9 (75.0)	0.554
Clindamycin	3 (50.0)	6 (75.0)	0.334
TMP_SMX	18 (43.9)	30 (61.2)	0.101
Nalidixic acid	14 (38.9)	18 (45.0)	0.590
Norfloxacin	9 (42.9)	13 (68.4)	0.105
Ofloxacin	6 (20.0)	10 (29.4)	0.411
Ciprofloxacin	10 (27.0)	14 (33.3)	0.489
Levofloxacin	7 (28.0)	7 (28.0)	1.000
Moxifloxacin	1 (50.0)	–	–
Nitrofurantoin	3 (23.1)	3 (18.8)	0.775
Fusidic acid	3 (50.0)	7 (87.5)	0.124
Rifampin	2 (33.3)	5 (62.5)	0.280
Vancomycin	3 (25.0)	1 (8.3)	0.273
Teicoplanin	3 (25.0)	0 (0.0)	0.064
Fosfomycin	4 (9.1)	11 (20.8)	0.114
Linezolid	0 (0.0)	0 (0.0)	
Amphotericin B	0 (0.0)	0 (0.0)	
Caspofungin	3 (20.0)	2 (11.1)	0.085
Mycafungin	0 (0.0)	0 (0.0)	
Fluconazole	9 (60.0)	4 (22.2)	0.027
Voriconazole	0 (0.0)	0 (0.0)	
Flucytosine	–	2 (40.0)	–

Concerning anti-fungals, when looking at total *Candida* species (*albicans* and non-*albicans*), a statistically significant decrease in resistance to fluconazole is detected (60% resistance pre-explosion and 22.2% resistance post-explosion (p-value = 0.027). The number of *Candida albicans* post- explosion. Has increased which are susceptible to fluconazole. Prior to the explosion, two out of five species of candida excluding glabrata were resistant to fluconazole; however, one out of twelve of candida species excluding glabrata were resistant to fluconazole (*P* = 0.022). Despite the emergence of new non-albicans species post-explosion, their number compared to albicans species was low. There was no significant change in the incidence of the different *Candida* species. Out of total candida species, the rate of albicans and tropicalis was higher post explosion compared to pre-explosion, 75% vs 62.5% respectively, with a non-statistically significant *p*-value of 0.99.

## Discussion

The rate of HAI did not differ between both groups, however, there was a remarkable difference between the rate of MDROs pre and post-explosion. The use of broad-spectrum antibiotics empirically in both groups could be justified with the suspicion of HAI. The increase in the number of colonization cases in the post-explosion group could have affected the hospital microbial flora. For instance, *E.coli* resistance was higher post-explosion. The choice of pre-operative antibiotic prophylaxis used before and after the explosion did not change; hence, the trend in the increased resistance of acquired microorganisms in hospital settings could be directly associated with the explosive event. The admission of Covid-19 patients post-explosion also might have played a role in affecting our antibiotics choice. To note, the first case of Covid-19 was admitted to the hospital on August 6th, which is after the Beirut explosion. A total of 241 cases were admitted till December 31st 2020.

A retrospective study examined the clinical impact of MDROs arising in one US military treatment facility post war in Iraq and Afghanistan [4]. The study results showed that the incidence of MDR ABC infection increased from 2.3% in 2001 to 11.9% in 2005 at the US Army Institute of Surgical Research. Most of the ABC isolates had broad spectrum antimicrobial resistance with mortality rate of 22% in the infected patients in contrast to 7.7% in those without infection. Other notable gram-negative bacteria infecting combat casualties in these US facilities included MDR *P. aeruginosa* and *Klebsiella pneumonia* [4].

Furthermore, as a result of the Southeast Asia earthquake and tsunami in 2004, traumatic injuries to head, chest, and limbs were often contaminated with highly resistant bacteria, mainly gram negative MDROs, such as *P.aeruginosa*, *Enterobacter cloacae*, *Aeromonas* spp., *Proteus* spp., *Bacteroides* spp., *Acinetobacter baumanii* (MDR) and *E.coli* (ESBL) [14].

Findings from the 1999 Marmara earthquake showed that HAI with MDROs was significantly increased. The following potential pathogens were found in the cultures: *A. baumannii*, MRSA, *P.aeruginosa*, *E.coli*, *K.pneumoniae*. All *Staphylococcus aureus* strains were found to be resistant to methicillin in vitro. Two strains of *A. baumannii* and one *P. aeruginosa* were found to be resistant to all antimicrobials including carbapenems [15]. In our study, a significant difference was found between the rate of *Stenotrophomonas maltophilia* between the pre- and post-explosion groups. However, it is important to note the increase in the use of carbapenem during COVID-19 surge, coincided with the post-explosion period [16]. This pandemic and its associated use of broad-spectrum antimicrobials could have also interfered with the outcomes such as the emergence of new *Candida* species. Also, a systematic review presenting antimicrobial resistance emergence in the Middle East showed that gram negative resistance was highly emerging with carbapenem resistance. *Acinetobacter spp* at 74.2%, and ESBL producing *Enterobacteriaceae species* (*K. pneumoniae* and *E. coli*) at 60.2%. These data were clearly noticed in our study results, with an increase rate of ESBL post-explosion [14].

To take this further, the emergence of higher numbers of carbapenem resistant *Enterobacteriacae* (CRE) after the events of August 4th raised several questions related to the rationale behind this increase in resistance. For example, contributing factors could be due to exogenous bacteria from the environment causing more resistance in our hospital, or heavy use of antibiotics after the explosion due to deep and life threatening injuries, or whether it was a confounding factor related to the COVID-19 pandemic where broad spectrum antimicrobials were used for treatment of COVID-19 bacterial complications especially that the first Covid-19 patient admitted to our hospital took place after the explosion. It is important to note that the explosion happened within the same period of the COVID-19 pandemic.

In terms of increasing the prevalence of bacterial resistance, post Marmara earthquake in 1999, the mortality rate was significantly higher in those patients with HAI (34.1%) than in those without HAI (1.7%) 48 h post hospitalization (*P*-value< 0.05) [17].

During the Syrian conflict, clinician researchers were alarmed with the increase in antimicrobial resistance, especially with the overwhelmingly crowded and unhygienic environment, the increase in the number of injuries, the financial sanctions and the decrease in medical resources [18]. Another study, conducted in Afghanistan, explained the crucial role of infection control measures, laboratory diagnostic capabilities, and active surveillance of antimicrobial resistance as a mean to limit the emergence of antimicrobial resistance post-conflicts [19]. In the recent ongoing conflict, in Ukraine, the emergence of antimicrobial resistance are reported in hospitals [20].

Our study has several limitations. First, our medical center is a 138-bed university hospital located in metropolitan Beirut, surrounded by other hospitals with close proximity, which limited the number of patients that could have been included. Second, since no cultures were collected at the day of the explosion, the investigators had to consider this group of patients as a control group, which decreased the number of the patients included. Third, the explosion occurred amidst the COVID-19 pandemic, where many patients were hospitalized with complications and required treatment with broad-spectrum antimicrobials.

## Conclusion

To our knowledge, this is one of the few studies that compare the flora changes in a hospital after an explosion, war conflicts or a natural disaster. The rate of growing MDROs increased post the explosion at a university hospital that received a mass casualty the day of the explosion. We could not identify a significant difference in the type of microorganisms. However, new emerging microbes were seen post-explosion mainly *Candida* species.

## Data Availability

The data analyzed during the current study are available in the Infection control repository at the Lebanese American University Medical Center- Rizk Hospital (LAUMC-RH) and are available upon a request to the corresponding author.

## Abbreviations

LAUMC-RH: Lebanese American University Medical Center- Rizk Hospital
NI: Nosocomial infections
MDRO: Multi-drug resistant organism
ABC: *Acinetobacter baumaunni calcoaceticus* complex
CDC: Center for disease control and prevention
MRSA: Methicillin resistant *staphylococcus aureus*
VRE: Vancomycin resistant *enterococci*
MDR: Multidrug resistant
ESBL: Extended spectrum b-lactamase producing *enterobacteriacae*
HAI: Hospital acquired infections
CSF: Cerebrospinal fluid
E.coli: *Escherichia coli*
CRE: Carbapenem resistant *enterobacteriacae*
